# Teacher sensitivity as a bridge to emotion regulation for students with special educational needs in their emotional and social development in physical education

**DOI:** 10.3389/fspor.2025.1671290

**Published:** 2025-11-04

**Authors:** Leefke Brunssen, Valerie Kastrup

**Affiliations:** Department of Sports Science, Bielefeld University, Bielefeld, Germany

**Keywords:** teacher-student relationships (TSR), special educational needs (SEN), emotional and social development (ESD), physical education (PE), teacher sensitivity, emotion regulation (ER)

## Abstract

**Introduction:**

Students with special educational needs in their emotional and social development (SEN-ESD) often experience strained teacher-student relationships (TSR). Physical Education (PE) presents a dual-natured context: while offering explicit curricular socioemotional learning opportunities, its embodied interactions and open setting may feel overwhelming for these students. Cross-disciplinary research on SEN-ESD suggests scarcity of qualitative work centering student and secondary teacher voices concerning TSR. Guided by attachment theory, this qualitative study investigated: (1) how students with SEN-ESD and PE teachers perceive the affective quality of their TSR in inclusive PE settings, and (2) what concepts are related to the perceived affective quality of TSR.

**Materials and methods:**

Using Grounded Theory, we conducted and analyzed semi-structured interviews iteratively with 22 students (ages 10–16) with formal SEN-ESD diagnoses and 18 PE teachers at German regular secondary schools until theoretical saturation was achieved.

**Results:**

Analysis revealed three interrelated dimensions: (1) the category perceived TSR quality (conflict ↔ closeness); (2) the related concept teacher sensitivity (low ↔ high); (3) the related concept students' emotion regulation strategies (dysfunctional ↔ functional).

**Discussion:**

Analysis of the six emergent patterns reveals teacher sensitivity as the pivotal factor shaping teacher-student relational dynamics. Co-constructed agreements foster a secure base for students, supporting functional emotion regulation, whereas rigid rule-enforcement perpetuates cycles of marginalization. Strikingly, some students rationalized aggression as a subjectively functional strategy (e.g., enforcing reciprocity fairness), clashing with systemic norms. Ultimately, the embodied context of PE emerges as a dual-natured relational microcosm: it can offer socioemotional growth when teacher sensitivity is high, but carries escalation risks when subjective-normative discrepancies remain unaddressed.

## 1 Introduction

Extensive research underscores the critical role of teacher-student relationships (TSR) in fostering academic progress and social-emotional well-being among students with special educational needs (SEN) in their emotional and social development (ESD). Notably, students with externalizing behaviors and special educational needs in their emotional and social development (hereafter SEN-ESD) and their teachers report more strained relationships compared to typically developing peers (1). For students with SEN-ESD, positive dyadic interactions with teachers are particularly important, because they often struggle to initiate and maintain relationships (2).

Physical Education (PE) introduces unique relational dynamics. Unlike traditional classrooms, PE curricula explicitly emphasize social-emotional skill development (e.g., 3, 4), providing opportunities to improve cooperation, interaction, and conflict resolution through embodied, interactive activities. However, these same conditions—intense peer interactions, competition, physicality, and sensory stimuli—require social competencies that students with SEN-ESD may find overwhelming (5). This dual-edged nature may position PE as both a transformative space for social-emotional growth and a high-stakes environment where inadequate support reinforces marginalization.

Despite acknowledging the significance of teacher-student relationships (TSR) for students with SEN-ESD, *three research gaps remain* salient. First, existing scholarship predominantly employs correlational designs and teacher-reported data; to our knowledge, no qualitative study examines how teachers and students co-construct TSR in practice (1). Second, while preschool/elementary settings feature prominently in research, secondary education contexts—and their distinct relational dynamics—remain underexplored (6). Third, no work investigates how PE's unique environment, inherently characterized by frequent embodied interactions, shapes TSR development or to what extent its explicit social-emotional curricular focus creates distinctive pathways for socio-emotional growth (7). This gap constitutes a significant oversight given these students' heightened dependence on teacher support for relationship initiation and maintenance (2).

Addressing these gaps, this study examines TSR perceptions between PE teachers and students formally identified with SEN-ESD (in German “Förderschwerpunkt emotionale und soziale Entwicklung”)^1^—who were described by their educators as exhibiting externalizing behaviors—in German general schools. To understand these relational dynamics, we employed Grounded Theory methodology integrated with an attachment theory perspective centered on TSR processes for students with Emotional and Behavioral Problems^2^ (EBP) in school contexts.

### 1.1 Externalizing SEN-ESD: conceptualization and implications

Empirically, EBPs are classified into externalizing and internalizing domains (8). Externalizing problems manifest as outwardly directed behaviors such as oppositional defiance, aggression, delinquency, hyperactivity or inattentiveness, whereas internalizing problems are characterized by inwardly directed symptoms such as anxiety, sadness, depression or social withdrawal (5, 9). These behaviors can be conceptualized in three ways: (1) as mental health disorders diagnosed via criteria in systems like the DSM-5 (9) or ICD-11 (10); (2) as psychosocial problems assessed through dimensional taxonomies quantifying symptom severity; or (3) as SEN in the area of social-emotional development, requiring tailored educational support^3^. These three conceptualizations may coexist or manifest independently (5). In Germany, the identification of students with a SEN-ESD is governed by state-specific educational laws (“Schulgesetze der Bundesländer”) and the guidelines of the *Standing Conference of the Ministers of Education and Cultural Affairs* (3). To understand how TSRs can mitigate social-emotional and academic risks, attachment theory explains the relational dynamics shaping students' behavioral and emotional development.

### 1.2 Attachment theory and teacher-student relationships

According to Bowlby's (11, 12) foundational attachment theory, humans inherently seek emotional bonds from birth. These bonds are organized through an attachment behavioral system activated during distress when children seek caregiver proximity. Ainsworth et al. (13) empirically validated and expanded Bowlby's framework, demonstrating that responsive care deactivates attachment behaviors while enabling exploration in safe environments. Early relational experiences form Bowlby's concept of internal working models (2, 11) that shape caregiver expectations, self-perception, emotional regulation, and lifelong relationship patterns. Critically, these mental representations automatically guide interpretations of partner behaviors, yet “teachers are not always aware of their thoughts and feelings […], which may be more implicit than explicit” (6). As Spilt et al. (6) note, while explicit cognitions can be measured through direct methods (e.g., questionnaires), capturing implicit processes requires indirect approaches (e.g., narrative interviews)—a methodological distinction, which is unapplied in TSR research. These models manifest as secure or insecure attachment styles (14), with insecure attachment (characterized by anxious-resistant hyperactivation or avoidant deactivation of attachment needs) impairing emotion regulation and development (15).

Given these lifelong implications of early attachment patterns, understanding how they operate in school settings requires a relational framework that accounts for dynamic teacher-student exchanges. This is provided by *Developmental Systems Theory (DST)*, which conceptualizes TSRs as “dyadic microsystems” characterized by dynamic interplay between intrapersonal, interpersonal, and contextual factors (16). Four key components define this microsystem: First, characteristics of the teacher and student (e.g., attachment histories, temperament and self-regulation ability); second, the classroom and school environment; third, processes of information exchange during daily interactions; fourth cognitive-affective mental representations of self, others, and relationships (16). Thus, students enter school with diverse attachment representations shaped by their early experiences, influencing their expectations of teachers as secondary attachment figures (2).

Additionally, teacher-student relationship representations act as cognitive filters that shape interpretations and expectations, operating as *self-fulfilling prophecies* (17). Challenging classroom behaviors stemming from impairments in emotional regulation such as aggression or withdrawal may be misinterpreted by teachers as rejection or defiance, triggering punitive responses that reinforce conflict cycles (18). Conversely, students internalizing relational distance often overlook relationship-enhancing cues. Stable TSRs may disrupt these patterns, serving as protective buffers (19).

Guided by the attachment perspective, TSRs are multidimensional constructs encompassing interpersonal behaviors and feelings of teachers and students. The *secure base* metaphor describes supportive behaviors enabling exploration, while the *safe haven* metaphor reflects care during distress (20). *Three key dimensions of TSRs* are *closeness*, *conflict*, and *dependency*. *Closeness* refers to warmth, positive affect, open communication, and trust, indicating that the teacher is attuned to the child's needs while the child feels comfortable approaching the teacher and uses them as a secure base and safe haven. *Conflict* refers to negativity, lack of rapport, and conflicted interactions such as quarrels, reflecting an unpredictable, unreliable, or hostile relationship where the child cannot rely on the teacher as a secure base or safe haven. *Dependency* reflects age-inappropriate possessive or clingy behaviors, indicating the child excessively and ineffectively uses the teacher as a secure base and safe haven (20).

Research shows that for students with SEN-ESD, conflict within TSR predicts externalizing problems, while dependency predicts internalizing problems (21). Additionally, high-quality TSRs correlate with fewer behavioral problems and greater prosocial behaviors and academic success, whereas low-quality TSRs relate to increased difficulties (1). Notably, students with SEN-ESD rate the perceived dimension of conflict higher than teachers, while closeness ratings show no significant divergence (21, 22).

*Teacher sensitivity* critically influences TSR quality, encompassing educators' capacity to respond *sensitively, timely*, and *appropriately* to student behaviors while anticipating needs and emotions. This fosters students' autonomy and sense of belonging (18). From an attachment perspective, sensitive teachers function as *ad hoc* attachment figures, offering a safe haven and secure base that enables students to explore environments and manage academic demands, thereby shaping learning behaviors (17). A meta-analysis (23) affirms sensitivity as a key predictor of TSR quality, especially for students with social-emotional difficulties.

Attachment representations shape students' behavioral *emotion regulation strategies*. In general, these strategies are broadly categorized as either adaptive or maladaptive based on their long-term effectiveness (24). Critically, insecure attachment directly correlates with impaired regulation (11) but also with heightened reliance on maladaptive or dysfunctional strategies (15). Defining this core mechanism, Thompson (25) characterizes emotion regulation as “the extrinsic and intrinsic processes responsible for monitoring, evaluating, and modifying emotional reactions, especially their intensive and temporal features, to accomplish one's goals.” Such strategies can occur *internally* (e.g., cognitive reframing) and *externally* (e.g., physical activities), categorized as *functional—*processing and maintaining emotions—and *dysfunctional*—blocking or rejecting emotions—strategies (24, 26, 27).

### 1.3 The present study

While attachment theory illuminates how implicit mental representations shape teacher-student interactions, no qualitative study has examined TSRs from both teacher and student perspectives for students with SEN-ESD. This study addresses that gap by applying the framework to Physical Education—a context defined by embodied interactions and explicit socio-emotional demands—asking:

1. How do students with special educational needs (SEN) in their emotional and social development (ESD) and Physical Education (PE) teachers perceive their Teacher-Student Relationship (TSR) in inclusive PE settings?
2. What concepts are related to the perceived affective quality of TSR between students with SEN-ESD and PE teachers in inclusive PE?

## 2 Materials and methods

This study employed a Grounded Theory approach following Corbin and Strauss (28), augmented by Reflexive Grounded Theory principles (29), to explore inductively emerging themes in inclusive PE for students with SEN-ESD through constant comparative analysis. Ethical approval was obtained from the institutional review board (Protocols 2024-047 for PE teachers; 2024-048 for students).

Consistent with Spilt et al.'s (6) statement for accessing implicit processes, we employed qualitative, semi-structured interviews to avoid reliance on explicit self-reports that may overlook unconscious relational dynamics. An overall number of 18 PE teachers (coded T1–T18, where “T” = teacher) and 22 students (coded S1–S22, where “S” = student) with SEN-ESD (ages 10–16) from general education schools in three German states (North Rhine-Westphalia, Lower Saxony, Bremen) were interviewed between November 2023 and April 2025. At the time of each interview, all students were formally diagnosed with SEN-ESD within the German education system and described by their educators as exhibiting externalizing behaviors. None of the students was diagnosed with autism spectrum disorder. All teachers were general, not special education teachers and taught at least one student with SEN-ESD in PE at the moment of the interview. No relationship existed between teachers and students.

After participants had described their most recent PE session, all were asked to recount a memorable situation from PE. Teachers were specifically prompted to focus on a memorable situation involving a student with SEN-ESD they currently taught in PE. All teachers and some students then described a detailed story about a particular situation in a narrative style. Follow-up questions probed details relevant to the participant's narrative. Notably, nearly all participants initially described a conflict-laden situation when asked for a memorable situation. The interview protocol then explicitly invited participants to recount another positively as well as challengingly perceived situation. The interviews were audio-recorded, transcribed verbatim, and anonymized. MAXQDA 24 software (30) was used for code management and ensured systematic comparison across interviews and memos. Participant quotes are identified by their code (e.g., S1 for Student 1, T3 for Teacher 3) followed by a position number (Pos.), which corresponds to one sequential speaker turn in the transcript.

**Figure 1:**
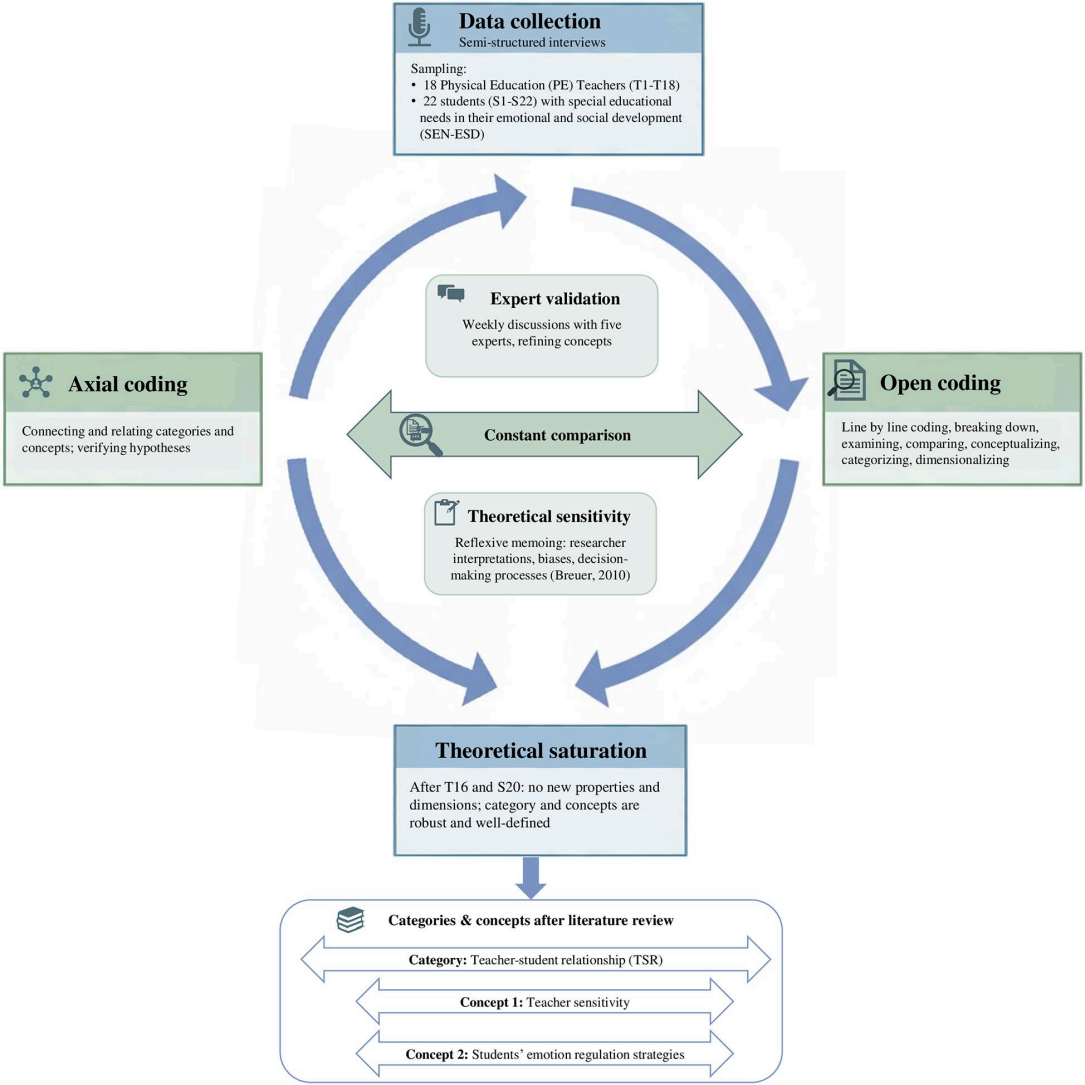
Grounded theory research process following Corbin & Strauss (2008).

The analytical process (Figure 1), guided by the principles of theoretical sampling, proceeded iteratively. First, an initial sample of 10 interviews was conducted with participants from each of the two pre-defined groups. Open coding fractured interview transcripts line-by-line to identify provisional concepts. Following the initial open coding of early interviews, we systematically sought to maximize variation in the emerging concepts. The interview displaying the strongest contrasts was then selected for subsequent open coding. After 4–5 interviews per group, we started alternating between open and axial coding. Axial coding involved grouping concepts, relating and dimensionalizing them, which led to the development of a coding paradigm for understanding the data from both groups. As concepts became more numerous and more abstract, some formed higher-level, more abstract categories. Concepts and categories are generated in the same analytic process through constant comparison. Through this process, the category *Teacher-student relationship (TSR)* was developed. The concepts of *Teacher support* and *Students' conflict resolution strategies* were identified as being closely related to TSR, because they are influenced by the perceived quality of TSR.

Consistent with the principles of theoretical sampling (28), the subsequent process focused on sampling concepts, their properties, dimensions, and variations rather than specific groups of individuals. New interviews were then conducted in an iterative process, with follow-up questions targeting themes identified as relevant in prior analyses. For example, early data indicated that rules were a relevant topic for both groups; consequently, subsequent interview guides were amended to include probes such as: “What role did rules play in this situation?”.

This process of dimensionalizing involved breaking down the properties of our categories and concepts to specify their full range of dimensions. For instance, the category TSR has the property quality, which was dimensionalized as ranging from low to high. This iterative process of verifying relationships and dimensionalizing properties through constant comparison continued until theoretical saturation was achieved, which was indicated by no new properties or dimensions emerging and the categories and concepts being robust and well-defined. In this process, different patterns of TSR were identified to account for variations. These patterns were synthesized by cross-mapping participants' shared positions across the dimensions. After the 16th PE teacher interview and the 20th student interview, the final two interviews with each group (T17, T18, S21, S22) yielded no new codes, properties, or insights into categories (28). Theoretical sensitivity was maintained through reflexive memos documenting researcher interpretations, biases, and decision-making processes (29).

The analytical process was strengthened through a rigorous validation procedure. Following Corbin and Strauss's (28) canons and procedures, the corresponding researcher engaged in weekly two-hour interdisciplinary discussions with four other Grounded Theory experts to test concepts and guard against bias. In these sessions, a raw data excerpt was individually coded and interpreted by each member. This was followed by a collaborative discussion of these interpretations, during which the principal researcher remained silent. The process of collaborative constant comparison challenged initial analyses and led to refinements in conceptual precision. For instance, the group challenged our initial normative analysis of certain conflict resolution strategies (e.g., aggression) as “dysfunctional”, arguing that from some students' subjective perspective, these strategies were experienced as functional. This consensus led to a reconceptualization, which later also developed into a central pattern (Pattern 6) in the findings. Trustworthiness was further ensured through triangulation of interview data with reflexive memos (28).

As interviews were conducted in German, the quotes used in this study were translated as accurately and directly as possible by the first author, who has an academic background in English language teaching. To ensure semantic accuracy, quotations were back-translated into German and cross-checked against the original transcripts. Formal member-checking with students was ethically precluded to prevent stigmatization. Member-checking with PE teachers was conducted as the theoretical framework and results were presented and discussed with seven practicing PE teachers. They reviewed the findings for plausibility and confirmed that the identified patterns and dimensions resonated with their professional experiences in the field.

## 3 Results

Analysis of all interviews reveals that *contextual conditions* shape teacher-student relationships (TSR) quality by presenting both opportunities and challenges, as reported by students with special educational needs in their emotional and social development (SEN-ESD) and their Physical Education (PE) teachers. A key factor is students' *high motivation* for physical activity and especially team games. Many students describe PE as their favorite subject and emphasize its psychophysical necessity: “I need the movement” (S9, Pos. 58).

At the same time, PE is viewed by both students and teachers as a critical arena for social competence development, a subject unlike any other where social skills “take center stage” through the “process of engaging with others and occasionally stepping back” (T12, Pos. 26). However, both students and teachers consistently report more conflicts in PE compared to the classroom. S20 recounts: “In PE, we're always losing it, and it's really rare for us to argue in the classroom. But in PE? ALWAYS. […] People get insulted, spat on, hit with balls.” (S20, Pos. 142). Reasons cited for frequent conflicts include the open environment, sensory demands, safety risks, and competitive activities. As one teacher explains: “In PE, everything just comes together—ambient noise, classmates, […] team sports, […] social interaction” (T5, Pos. 8). Students echo this, linking competitions to conflict: “Yeah, all the time. Because we always play against each other, and then there's always insults, fights, and stuff” (S20, Pos. 138). Paradoxically, despite these tensions, almost all students prefer team sports and games—the very contexts they associate most conflict-laden. This duality frames PE as both a space for embodied, *collaborative learning* and a *potential catalyst for emotional escalation*.

### 3.1 Category TSR and related concepts

**Figure 2:**
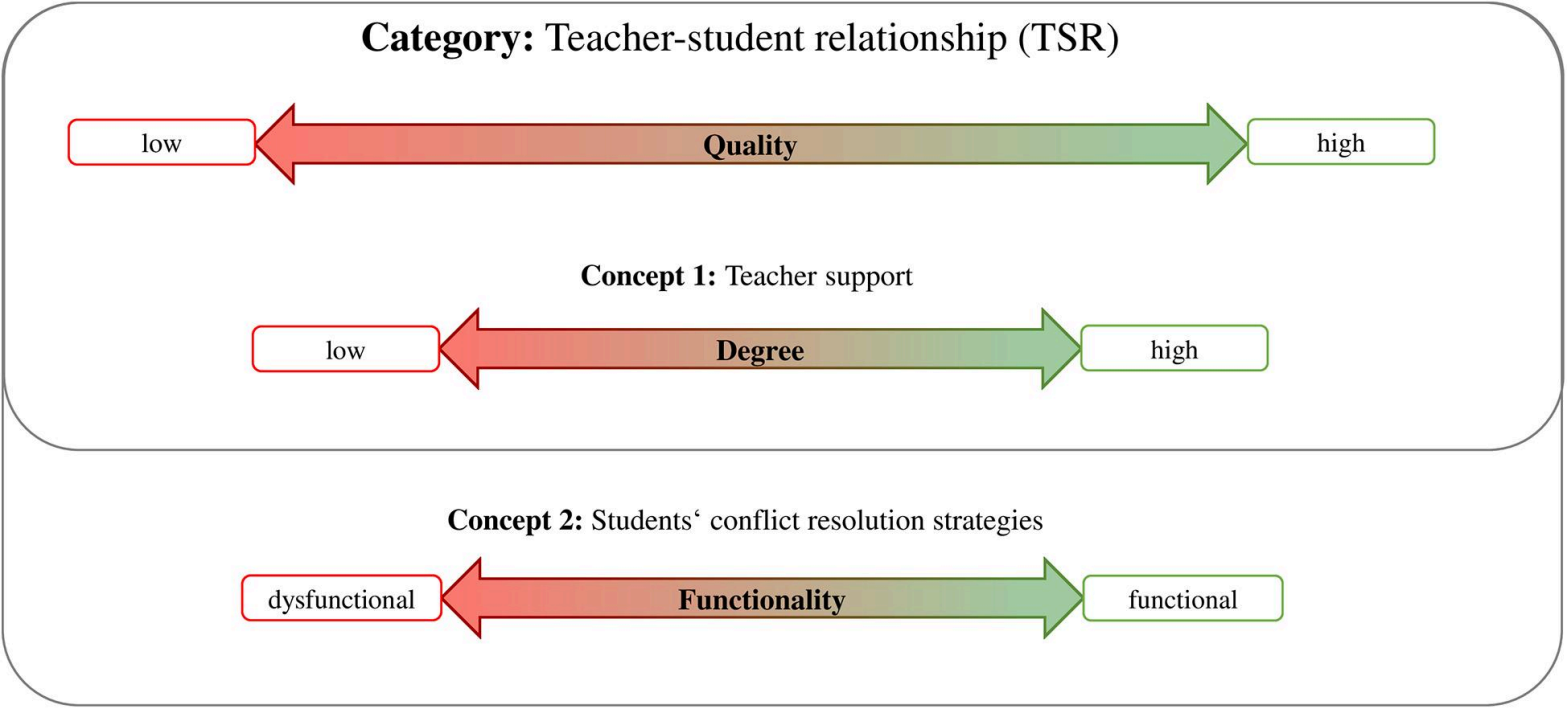
Dimensions and properties of the category teacher-student relationship (TSR) and related concepts.

The analysis of the *category* TSR in inclusive PE settings revealed two interconnected *concepts* shaping relational dynamics in both groups. Subsequently, properties and variations (28) that were assigned to each category or concept will be described as well as visualized in Figure 2. (1) The category of reconstructed *TSR* from the perspective of the interviewed student or PE teacher has the property of *quality* and is dimensionalized as ranging from *low* to *high*; (2) the concept of reconstructed *teacher support* in the described situation of the interviewed teacher or the perceived teacher support of the interviewed student has the property of *degree* on a dimension from *low* to *high*; and (3) the concept of *students' conflict resolution strategies* in conflictual situations in the described memorable situation from the perspective of both groups has the property of *functionality* and is dimensionalized as ranging from subjectively *dysfunctional* to subjectively *functional*.

### 3.2 Category TSR quality and concept 1 PE teacher support

**Figure 3:**
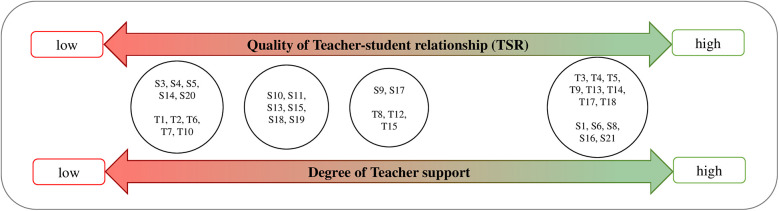
Dimensionalizing physical education (PE) teacher-Student Relationship (TSR) and Teacher Support.

A core finding of this study is that the perceived TSR quality and PE teacher support are so closely interrelated they can be *conceptualized together*. The second concept of students' conflict resolution strategies is then examined in relation to this framework in section “3.3 Concept 2: Subjective Functionality of Students' Conflict Resolution Strategies”. The following Figure 3 presents variations in TSR quality across participants.

#### 3.2.1 High TSR quality and high PE teacher support

(Seven teachers; Five students).

A high relationship quality is characterized by *responsive support as the foundation of safety*. This is grounded in teachers' *situational awareness*, enabling them to detect students' stress signals early and ensure physical and emotional safety through de-escalation. Central to this is constant awareness in tense interactions, requiring immediate intervention and “radiating calmness” (T9, Pos. 24). Students value such rapid support, noting how teachers: “stopped him right away.” (S8, Pos. 99). This support also manifests in proactive support: “You have to see the kids, be in touch with them, and somehow say, ‘I notice this isn't working—how can I help you?’” (T3, Pos. 21).

High quality relationships further feature *transparent agreements and predictability*. These *preventively communicated rules* establish a predictable climate, reducing conflicts. Teachers note that clear agreements foster student gratitude for structure (T3, Pos. 21). Students perceive this predictability as relieving, as it provides security in potentially overwhelming situations: “Our agreement was that when I'm not doing so well, I let him know. Then I can go out. […] I stay within the sports hall. […] when I feel better again, then I can come back” (S21, Pos. 44). This structured flexibility balances autonomy with boundaries, institutionalizing needs while strengthening trust.

Positively perceived relationships are also characterized by *dialogic communication on equal footing*, where teachers *recognize subjective realities*, clarifying perceptual discrepancies. A teacher reflects on the need to reconcile different views of fairness: “children see it differently […] you have to clarify it with them first” (T3, Pos. 25). Students experience this dialogue as validation of their perspectives—“it's not like she just says: ‘that's nonsense’” (S1, Pos. 70)—which fosters trust and positive relationships. Involving students as *equal actors* emerges as a key aspect of high TSR quality. Intentional inclusivity appears in practices where teachers actively ensure all students are seen and supported: “Our new PE teacher, he really pays attention to EVERYONE^4^” (S21, Pos. 36). The emphasis on “EVERYONE” contrasts with prior exclusion, while now tailored support “You can take your TIME” (S21, Pos. 36) fosters agency and attempts at new tasks, reflecting a balance between autonomy and structured support.

#### 3.2.2 Ambivalent TSR quality and PE teacher support

(Three teachers; Two students).

A sharp tension *between empathic attunement and structural rigidity* characterizes these teachers' practice. While T15 demonstrates cognitive empathy, metaphorically framing students' emotional states “they're in their tunnel” (Pos. 28) and rejecting personal blame, the teacher simultaneously enforces standardized disciplinary protocols during conflicts (Pos. 28). This duality is rationalized through an imperative to “somehow try to build a relationship. Nevertheless, the regulatory structures must be enforced” (Pos. 32). Prioritized relational efforts clash with *uniform sanctions*, such as red cards, lesson exclusion and standardized penalty catalogs during rule violations or competitive stress. This rigidity persists despite partial post-conflict resolution from the students' perspective: “later he talks to us and then it is usually clarified” (S17, Pos. 142).

#### 3.2.3 Low TSR quality and low PE teacher support

(Five teachers; 11 students).

*Teachers* whose perceived TSR quality in the “memorable situation” was reconstructed as negative exhibit a pedagogical ethos prioritizing *structural control over individualized need* orientation. T1's statement, “just because they have special educational needs, they still have to adhere to rules” (Pos. 23), exemplifies a normative compliance framework that operates independently of individual support requirements. This universal behavioral expectation, framed as a logic of equal treatment, marginalizes individualized needs, as illustrated by T1's prioritization of collective instructional progress: “If this student wasn't here, we'd have more time for others in class” (T1, Pos. 103). Furthermore, relational dynamics follow a *transactional logic* that ties participation to behavioral conformity: “if they can behave according to the rules […] THEN they are very welcome” (T2, Pos. 126). The syntactic emphasis on “THEN” reduces pedagogical interaction to a reward contingency, reframing relationships as power negotiations. Authoritarian sanctions and metaphors like “keep [them] on a short leash” (T2, Pos. 25) further reveal asymmetrical dynamics that restrict exploratory behavior.

From *students' perspectives*, teachers' rule rigidity manifests in disempowering sanctions and emotional destabilization. S19 describes unilateral punishment without dialogue: “if there is a MISunderstanding, he JUST enforces a punishment […] He gets so ANgry […] you have no chance to express your view”. (S19, Pos. 35). This quote reveals the multi-layered nature of dysfunctional interaction patterns. The phrase “so ANgry” points to the teacher's emotional dysregulation, while the lexical choice “JUST” deconstructs a culture of arbitrariness. This non-recognition of agency is reinforced in S21's narrative: “He also showed no CONsideration for me. Sometimes he would say “Get OUT now’ or he would just call my mother, without even talking to me” (Pos. 38). For both students, the marginalization of their voice leaves them with a sense of powerlessness and at the mercy of authority.

The data on low TSR quality further reconstruct a *process of generalization*, *homogenizing and stigmatizing* all students with *SEN-ESD*. Teachers categorically attribute behaviors as “typical SEN-ESD”: “they're just completely stubborn. […] At the slightest conflict, they totally freak out” (T1, Pos. 19). These attributions ignore situational factors and frame behaviors inherent to a “disorder”. Students experience this generalization as *structural marginalization.* S20 describes bias in favor of “unremarkable” peers: “She didn't listen to both sides because […] I'm just the kind of person who often causes trouble” (Pos. 107). This *exclusion from conflict resolution processes* might intensify stigma internalization and deficit self-concepts.

### 3.3 Concept 2: functionality of students' conflict resolution strategies

The *Dimension* of the *second concept* ranges from perceived *functional* (self-efficacious, satisfying, or relieving) conflict resolution strategies of students ↔ to perceived *dysfunctional* (unsatisfying, associated with burden, escalation, powerlessness, or resignation) conflict resolution strategies of students.

**Figure 4:**
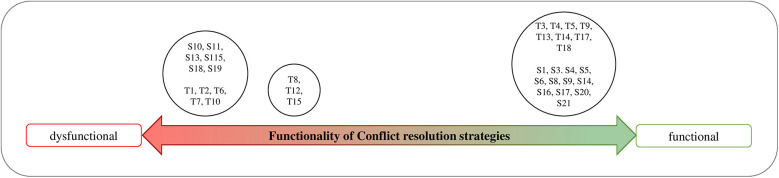
Dimensionalizing students' conflict resolution strategies.

The following Figure 4 presents the functionality of the resolution strategy used in the described situation.

#### 3.3.1 Functional conflict resolution strategies

(Eight teachers; Seven students).

For students with high and ambivalent perceived relationship quality toward their PE teachers, a differentiated repertoire of functional coping strategies can be reconstructed. These strategies are characterized by *cognitive reappraisal, proactive conflict resolution,* and *context-adaptive self-regulation*. S21's case exemplifies this as a biographical progression from maladaptive compulsions to increasingly resilient strategies through self-reflexion.

##### 3.3.1.1 Case example 1: causes of dysfunctional conflict resolution strategies and resilience development

S21's account reveals an early childhood socialization that, from the student's perspective, shaped dysfunctional coping patterns: “Even when I was REALLY young, I had major problems with aggression. […] I have a very impulsive family […] I never learned otherwise.” (S21, Pos. 46). These experiences in a violence-prone environment subjectively led to maladaptive behaviors, resulting in a vicious cycle of school sanctions and isolation. The dysfunction's intensity is underscored by S21's recall of frequently being collected from school in first grade due to an inability to regulate emotions: “I just didn't know how to hold back my emotions […] so that no one would get hurt.” (S21, Pos. 46). This inability to regulate emotions may reflect learned helplessness—where the absence of de-escalation role models solidifies *violence as a behavioral norm*. Crucially, the phrase “so no one would get hurt” signals a reflexive rejection of violence, marking a values shift toward social responsibility. S21's narrative thus reconstructs self-efficacy: adapting behavior to non-family contexts demonstrates resilience.

S21's account further illustrates a *graduated coping system* of acquired behavioral and regulation strategies utilizing internal and external resources: “By now, I've found my ways […] I just ignore it. When it gets too much, I tell teachers. […] If it doesn't get better, I tell my parents or take matters into my own hands” (S21, Pos. 54). This system entails first internal regulation (“ignore it”), second external support-seeking (“tell teachers or parents”), and third, self-efficacious confrontation. Even students with pronounced self-regulation skills encounters situations representing an *inevitable compulsion to act* where internal strategies fail: “I also notice myself when I'm doing something wrong. […] Sometimes I HAVE TO do that thing wrong, I'm sorry […] I couldn't do anything about it.’” (S21, Pos. 48). This reveals a paradox: despite metacognitive awareness of flawed actions, behavioral control is overridden, reconstructing social-emotional skills that are not (yet) developmentally age-appropriate—a characteristic frequently associated with students identified with SEN-ESD. The subsequent apology serves as a compensatory mechanism, acknowledging responsibility.

During uncontrolled externalization, *safer retreat spaces and distance* become critical. S21's agreement with her PE teacher allows her to independently leave the sports hall. Her narrative traces a developmental shift from maladaptive aggression “I needed someone to vent my aggression on” to functional strategies: “Now, I take a short walk, breathe deeply, and everything is okay again” (Pos. 50). Initially, aggression served as short-term affective release, perpetuating relational conflict. Now, self-initiated regulation offers three benefits: reduced physiological arousal via movement; cognitive restructuring through distancing and “deep breathing”; and social relief by preempting escalation.

*Physical and mental retreat options*, such as “punching a mat” (T13, Pos. 14) or a designated corner in the equipment room with “a chair and table to calm down” (T9, Pos. 24), are actively utilized by many PE teachers and are perceived by students (S1, S6, S21) as effective regulatory mechanisms. These *safer spaces* provide crucial support for *de-escalation and self-regulation* during emotionally stressful situations in PE. Furthermore, physical activity itself is valued as a critical outlet, “I need the movement” (S9, Pos. 58), while teachers contrastively emphasize its regulatory benefits over sedentary tasks: “having to sit still […] is incredibly difficult for them” (SL3, Pos. 21).

#### 3.3.2 Perceptual discrepancies: violence as functional conflict resolution

(Five students).

For some students with SEN-ESD with negatively perceived TSR, conflict resolution strategies that appear *dysfunctional from a systemic perspective* are *subjectively perceived as functional* and legitimate (S3, S4, S5, S14). This reflects a subjective understanding of normality where reciprocity is framed as fairness, *normalizing violence* through a logic of mutual exchange. As S3 asserts: “[I react] totally normal. […] If he insults me, I insult him back. If he pushes me, I push him back. If he hits me, I hit him too” (Pos. 61–63). This statement reflects an internalized understanding of normality that views violence as a norm, which—as in the earlier example from S21—may stem from biographical experiences. This reconstructed principle of equivalence, serving not intentional escalation but restoration of subjectively fair balance also legitimizes rule-breaking as retaliatory justice for perceived unfairness: “I hate Brennball^5^. But to really break the rules […], the ones others made. To break them, properly. […] The others did it too, tough luck” (S3, Pos. 107–109). The strategies chosen by students primarily serve immediate *emotional release* and coping with emotional overwhelm. *Violence* is not perceived as dysfunctional but as a logical and necessary response, providing short-term relief despite sanctions: “IF someone touches me, then I […] start FLIPPING OUT, and then […] I hit her in the face.” (S20, Pos. 190).

Students' actions clash with institutional norms, creating a *rift between subjective and systemic evaluations*. Students argue that in conflict-laden situations, *violence* ends the conflict and restores *fairness*. However, the system's interpretive logic frames *violence* as a *violation of rules*, a potential catalyst for escalation, and a trigger for sanctions. While sanctions aim to penalize rule-breaking, they reinforce students' perception of being treated unjustly.

#### 3.3.3 Dysfunctional conflict resolution strategies

(Eight teachers; Six students).

A pattern of *teacher neglect and student disempowerment* emerges where students, facing repeated conflicts and violence, adopt passive coping strategies stemming from absent or counterproductive support. S11 describes, “I'm the one who gets picked on […] then I run away” (S11, Pos. 27). Here, physical distancing serves as an external protective mechanism, though it risks weakening trust in their own agency. S18 explains: “I often get hit by my classmates […] I don't want that. But my PE teacher doesn't do anything about it. He thinks it's not a big deal […] I ignore it” (S18, Pos. 113). This statement crystallizes powerlessness into a *dual injury*: physical peer violence is legitimized by the teacher's passivity, as the violence is trivialized and not treated as a rule violation but as normality. The strategy “ignore it” may ease immediate conflict pressure but could reinforce long-term disempowerment—potentially leaving the student passive, distrustful of future support, and dependent on external regulation that does not occur.

A *boundary violation by a teacher* is exemplified in one case where the teacher's violent behavior contributes to a student's powerlessness: “Usually, it's normal to maybe get a warning. […] But he's the only teacher who immediately starts yelling and pulls on your T-shirt” (S19, Pos. 25). The physical boundary violation and verbal aggression, repeatedly emphasized in the conversation, create a climate of insecurity: instead of providing protection, the *teacher becomes a source of threat* and is experienced as an additional stressor.

##### 3.3.3.1 Case example 2: A prototypical escalation dynamic from a PE teacher's perspective

The interviews depict descriptions of conflict-laden situations in which regulatory patterns of students with SEN-ESD are exclusively perceived and categorized as dysfunctional (T8, T12, T15). This can be exemplified by T8's account of a cycle of emotional overwhelm and escalation. The conflict arises from a *trigger*: perceived humiliation and psychological vulnerability during a performance assessment. T8 recounts: “The student often felt put on display because he was stockier […] And whenever things didn't go the way he imagined, he'd flip out” (Pos. 12). The teacher identifies the emotional overwhelm within a context of physical exposure, perceived by the student as humiliation, yet makes no preventive adjustments, such as implementing protected formats or choice. Subsequently, verbal and physical aggression *externalizes the escalation*, further straining the relationship: “He chased after people, threw wooden objects at them, and even smashed them against the walls” (T8, Pos. 12). In the *post-escalation* phase T8 demonstrates care and willingness to engage in dialogue through a one-on-one conversation, yet this retrospective approach provides only temporary relief. The conflict cycle culminates in exclusion from PE classes: “*Twice excluded from participation”* (Pos. 12). T8's strategies remain confined to post-escalation strategies, reflecting a *control-oriented paradigmatic dominance* over students with SEN-ESD: “I show the affected students: ‘I'm watching, I see what you're doing’” (SL8, Pos. 14). This approach prioritizes disciplining but fails to address the underlying emotional overwhelm.

For *teachers,* who perceived their TSR quality as very low, similar conflict patterns emerged. However, the *absence of post-conflict dialogue* left conflicts unresolved and root causes unexamined. Lacking insight into contextual factors, these teachers framed students' conflict resolution strategies as significantly more dysfunctional than those of T8, T12, and T15, who perceived their TSR quality as ambivalent.

## 4 Discussion

The central findings of this study reveal that the perceived quality of the teacher-student relationships (TSR) in inclusive Physical Education (PE) with students with special educational needs in their emotional and social development (SEN-ESD) is decisively shaped by the degree of the teacher's support. These two aspects are strongly tied to the perceived functionality of conflict resolution strategies. Additionally, this qualitative analysis uncovers divergent implicit interpretive logics: some students subjectively assess normatively dysfunctional strategies—such as violence—as functional, which often collides with systemic logics of rule violations, leading to sanctions. These findings also underscore the importance of teacher sensitivity in PE's dual role as both a possible catalyst for conflict and a platform for social-emotional learning possibilities.

### 4.1 Empirical-Theoretical coherence of TSR

**Figure 5:**
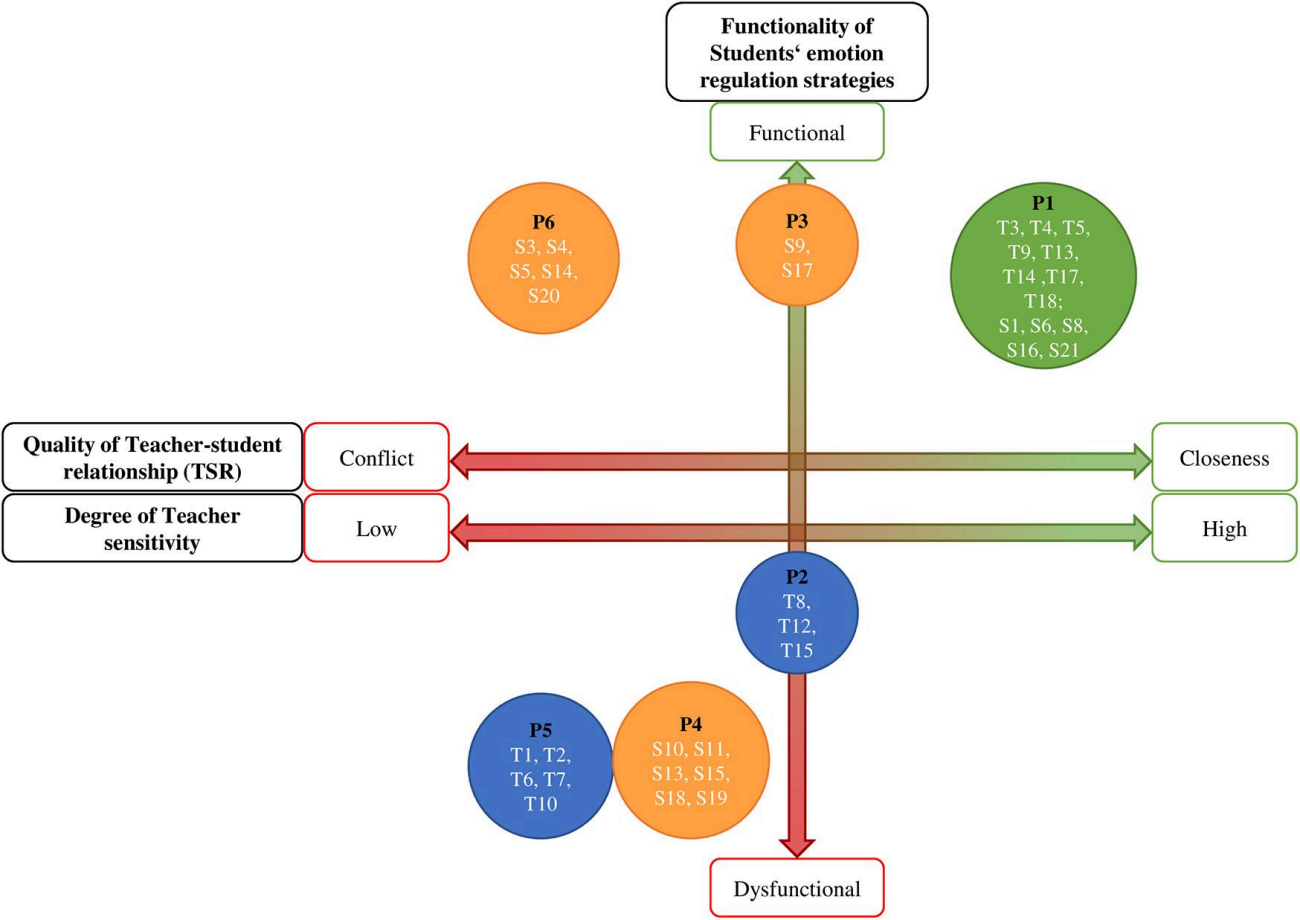
Mapping participants' perceptions onto the three dimensions with six emergent patterns (P1–P6) in inclusive physical education (PE) settings.

The qualitative data confirm that the TSR in inclusive PE with students with SEN-ESD can be contextualized through attachment theory and DST (16, 20) and can even extend the established dimensions of the TSR according to Pianta (20). As visualized in Figure 5, the three dimensions that emerged empirically from the data align closely with the theoretical framework:

1. The empirical category *TSR* quality corresponds with Pianta's (20) three *dimensions of TSR*. Consequently, the empirical dimension from “low” to “high” TSR quality is now conceptualized as the theoretical dimension from “conflictual” to “close” TSR quality.
2. The related, empirical concept *teacher support* mirrors *teacher sensitivity* (17, 18), emphasizing sensitive, timely, and context-appropriate manner.
3. The empirical concept of *conflict resolution strategies* maps onto *emotion regulation strategies* (26, 27), distinguishing adaptive (functional) from maladaptive (dysfunctional) coping.

Six distinct relational patterns (P1-P6) emerged from the data. They represent groups of participants who shared identical positions on both the *TSR quality* dimension (Figure 3) and the *Conflict Resolution*/*Emotion Regulation* dimension (Figure 4). These patterns and their relationship to the dimensions are elaborated below.

Students and teachers connect a close or conflictual TSR to the following: These six relational patterns collectively address our dual research questions (RQ): they reveal how students with SEN-ESD and teachers perceive TSR quality in inclusive PE (RQ1), while identifying teacher sensitivity and students' emotion regulation strategies as related concepts (RQ2). Grounded in attachment theory and DST, we now interpret the data's coherence with existing literature—examining consistencies, contradictions, and theoretical extensions—beginning with close TSRs, progressing to conflictual TSRs, then their relation to emotion regulation strategies, and concluding with PE's unique implications for TSR meaning.

#### 4.1.1 Close TSRs and high sensitivity

High teacher sensitivity often helped de-escalation in conflictual situations in *P1* (Table 1). P1 is the only pattern, in which students and teachers could be conceptualized together, largely because all *participants shared a common interpretive logic that precluded perceptual discrepancies*. This alignment appears reinforced by the high reflective competence observed among students in this pattern. Teachers who proactively recognized stress signals (e.g., interrupting conflicts promptly) and institutionalized structured autonomy (e.g., S21's negotiated “exit and return” rule) thereby created a *secure base* for students (20). Results show that this often enabled them to navigate challenges without feeling overwhelmed, supporting findings from the meta-analysis (23), identifying sensitivity as a key relational factor of TSR. As Vösgen-Nordloh et al. (1) demonstrate, TSR closeness correlates with fewer externalizing problems and greater prosocial behaviors and academic success. This study's results operationalize Pianta's closeness through trust-building actions—open communication (e.g., SL3's dialogue on “fairness”) and consistent attentiveness (e.g., S21 feeling “seen”)—which students equated with emotional safety, thereby aligning with and empirically grounding this protective function.

**Table 1:** Patterns of teacher-student relationships (TSR), teacher sensitivity, and emotion regulation strategies in inclusive PE.

Pattern, Name	Description	Participants	Exemplar Quotation
P1	In this pattern, five students and teachers perceived a close TSRs, a high teacher sensitivity and co-construct functional emotion regulation strategies through individualized agreements, fostering a secure base.	8 Teachers: T 3, 4, 5, 9, 13, 14, 17, 18	“I notice this isn't working—how can I help you?” (T3, Pos. 21)
Mutually Responsive Agreements			
		5 Students: S 1, 6, 8, 16, 21	
P2	In this pattern, three PE teachers perceive their relationships with the SEN-ESD student as ambivalent and describe the students' emotion regulation strategies as predominantly dysfunctional. Pedagogical efforts are confined to (mostly standardized) post-escalation interventions.	3 Teachers: T 8, 12, 15	“the regulatory structures must be enforced” (T15, Pos. 32)
Ambivalent TSR with Reactive Sanctions			
		0 Students	
P3	These two students employ subjectively functional strategies (e.g., self-initiated timeouts). By the students described pedagogical efforts of the PE teacher remain confined to post-escalation interventions. High self-reflective skills were reconstructed to compensate for ambivalent perceived TSR and teacher sensitivity.	0 Teachers	“I went and sat on the bench then […] they got in trouble then.” (S9, Pos. 286–288)
		2 Students: S 9, 17	
Autonomous Self-Regulation despite missing Teacher Sensitivity			
P4	The six students in this pattern perceive rather conflictual TSRs and low teacher sensitivity, employing internal dysfunctional emotion regulation strategies (e.g., suppression, acquiescence) to navigate distress. Systemic neglect (e.g., absent interventions) traps students in passive victimhood, while external strategies (e.g., S11's fleeing) fail to resolve systemic harm, perpetuating cycles of powerlessness.	0 Teachers	“if there is a MISunderstanding, he JUST enforces a punishment […] you have no chance to express your view.” (S19, Pos. 35)
		6 Students: S 10, 11, 13, 15, 18, 19	
Dysfunctional Coping in Systemic Neglect			
P5	These five teachers perceive a conflictual TSR and a low sensitivity was reconstructed in the described memorable situation. They mostly enforce rigid, rule-centered approaches (e.g., SL1's “regardless of special needs” rigidity) and essentialize behaviors as “typical SEN-ESD”. They often prioritize compliance over dialogue, which perpetuates conflict cycles and marginalization.	5 Teachers: T 1, 2, 6, 7, 10	“just because they have special educational needs, they still have to adhere to rules.” (T1, Pos. 23)
Conflict Cycles with Universal Sanctions			
		0 Students:	
P6	The five students in this pattern perceive a conflictual TSRs and low teacher sensitivity. They view strategies that are theoretically classified as dysfunctional (e.g., reciprocal violence) as subjectively functional.	0 Teachers	“I'm just the kind of person who often causes trouble.” (S20, Pos. 107)
Subjective-Normative Reciprocity Gap		5 Students: S 3, 4, 5, 14, 20	

This is a finding centrally anchored in Bowlby's (11) Attachment Behavioral System: In stressful situations, the attachment system of students with insecure attachments, which are statistically linked to SEN-ESD, can become activated. In this study, responsive behavior by the PE teacher (P1) seemed to de-escalate conflicts many times by serving as an *ad hoc attachment figure* (e.g., S21's PE teacher), redirecting energy toward exploratory behaviors and enabling the student to employ functional emotion regulation strategies—a finding that extends McGrath and van Bergen's (19) notion of stable relationships as protective factors by demonstrating their potential to *reverse* self-fulfilling cycles into positive feedback loops.

#### 4.1.2 Conflictual TSR and low sensitivity

In contrast, in rigid, rule-centered approaches (especially P4, P5, P6), teachers often perpetuated an insecure microsystem that can foster conflict escalation. This dynamic particularly affected students with lower self-reflection capacity, whose internalized working models appear to reinforce dysfunctional regulation patterns more frequently (11), which we often reconstructed for students in P6. Conflictual TSR perceptions in PE are often underpinned by low teacher sensitivity, aligning with Pianta's (20) *conflict* dimension and low sensitivity. Teachers who prioritized rule rigidity over individualized responsiveness (e.g., T1's assertion that students must adhere to norms “regardless of their special needs”) often fostered unpredictability and hostility, reflecting Pianta's characterization of conflict as a lack of rapport and reliability. This unpredictability might also stem from each students' different teachers in their different subjects; some who might differentiate behavior expectations as in P1 and some who mostly stick to generalized sanctions for all students as in P2/P5. Students here seemed to internalize this dynamic as emotional destabilization, describing arbitrary sanctions (e.g., S19's punishment without dialogue: “no chance to explain”) and affective teacher overreactions (e.g., S21's exclusion: “he just called my mom”), which mirror Pianta's observation that conflict correlates with adverse outcomes like disengagement.

This study reveals how teachers' misinterpretations of SEN-ESD behaviors—filtered through mental representations of rejection (17, 18)—fuel conflict cycles within TSR microsystems. Low sensitivity and rigid control amplified stigma and marginalization, as seen in P5 “Conflict Cycles with Universal Sanctions”: teachers essentialized behaviors as “typical SEN-ESD”, while some students internalized deviant labels (e.g., S20's “I'm seen as a troublemaker”). These dynamics align with finding that insensitivity can strengthen exclusion, replacing exploration with restriction (18). By foregrounding student voices, this study extends Pianta's conflict dimension, revealing how institutional practices (e.g., generalized sanction catalogues and essentialization) can compound relational harm and reinforce systemic marginalization.

Contrary to Pianta's (20) triad (closeness, conflict, dependency), this qualitative study revealed no evidence of age-inappropriate dependency in either group. This aligns with quantitative findings associating the dependency dimension with internalizing behavioral difficulties, whereas the focus of this study was on students with externalizing behavioral difficulties, which are connected with the *conflict* dimension (21).

#### 4.1.3 Relationship of TSR and emotion regulation strategies

The findings on the subjective functionality of conflict resolution strategies align closely with Emotion Regulation Theory (26, 27). For students with SEN-ESD, who frequently exhibit insecure attachment patterns (15), dysfunctional strategies are often reinforced, yet this study reveals critical nuances.

In *P1 “Mutually Responsive Agreements”*, both students and PE teachers perceived theoretically functional strategies as subjectively functional, provided physical outlet (external regulation), like T3's “punching a mat” and cognitive refraiming (internal regulation), like S21's “deep breathing”, particularly when tied to individualized agreements with the teacher.

In *P4 “Dysfunctional Coping in Systemic Neglect”*, students experiencing distress reported theoretically/subjectively internal dysfunctional strategies, such as S18's “helplessness through repeated acquiescence”, reflecting emotion suppression rather than processing.

Teachers in *P5 “Conflict Cycles with Universal Sanctions”* exclusively described dysfunctional, externalizing behaviors (e.g., “throwing wooden objects”), which in this study can be explained through their tendency to prioritize observable disruptions over asking students for their view, internal struggles or needs. This oversight is validated by students in *P5*, who urge teachers to “listen to both sides” (S20, Pos. 107), reconstructing the students' feeling of exclusion from the conflict resolution process.

Strikingly, some students in *P3 “Autonomous Self-Regulation despite missing Teacher Sensitivity”* described theoretically/subjectively functional strategies (e.g., self-initiated timeouts) despite perceived ambivalent TSR quality. This can be attributed to the reconstructed high self-reflective capacity and baseline in emotion regulation skills, which in this study seemed to reduced dependency on teacher sensitivity. This suggests that while teacher support is pivotal, individual competencies can buffer relational deficits. However, most students with SEN-ESD struggle precisely with these skills—making positive dyadic teacher interactions critical for their socioemotional development (2).

#### 4.1.4 Subjective-normative discrepancies

This study reveals subjective-normative discrepancies in how some students with SEN-ESD evaluate the functionality of emotion regulation strategies. Especially students in P6 *perceived normatively dysfunctional strategies* (e.g., using violence to resolve conflicts) as *subjectively functional*, interpreting reciprocity as a fairness restoration (e.g., aggression). These interpretive differences reflect a divergence in moral reasoning: a self-referential logic where fairness is defined as equivalent retaliation. Rather than amorality, students' endorsement of violence stems from a subjectively coherent system in which *reciprocity serves as both justification and goal*. Biographical exposure to aggression may reinforce this framework by normalizing violence as a restorative tool, creating dissonance between personal efficacy and normative ethical standards. Strikingly, in P1 “Mutually Responsive Agreements”, teachers consistently aligned with students' functionality criteria for conflict resolution. Here, no perceptual discrepancies emerged; instead, sensitive co-constructed agreements (e.g., negotiated “exit and return” rules) fostered mutual understanding of functional strategies, thereby strengthening close TSRs. These teachers in P1 also *individualized their behavioral expectations* and implemented *compensatory adjustments* for students who felt socioemotionally overwhelmed avoiding the labeling stigmatization seen in P5. This contrasted with P5 teachers, who applied universal classroom norms (e.g., SL1's rigid “regardless of special needs” approach), thereby exacerbating conflict cycles.

In this study, more students with SEN-ESD than teachers perceived their TSR conflictual, aligning with quantitative evidence of perceptual asymmetries in conflict ratings (21, 22). However, negative attachment representations from early experiences with primary attachment figures may predispose students to overlook teachers' positive cues offered in their role as secondary attachment figures. For teachers, establishing stable TSRs as *ad hoc* attachment figures can disrupt such patterns, acting as a protective buffer against relational erosion (19). Crucially, our qualitative lens reveals that divergent interpretive logics, evident specifically in students with conflictual TSR perceptions, constitute a previously unidentified source of discrepancy. This suggests that such logics may explain quantitative discrepancies found by van Loan and Garwood (22) and Vösgen et al. (21), where studies found alignment in closeness ratings but divergence in conflict evaluations.

#### 4.1.5 Implications: PE as an ambivalent space for social-emotional development

The PE context emerged as a room for risks and unique opportunities for students with SEN-ESD. Risks stem from inherent features like open environments, team dynamics, and sensory stimuli (T5: “loud noise, peer interactions”), which amplify emotional demands (S20: “In PE, we're always losing it”; T12: “conflicts occur more frequently”). Students with diagnosed needs in the area of SEN-ESD can face *double marginalization* if *conflictual TSRs* compound (1) PE's frequent conflictual interactions and (2) socioemotionally overwhelming situations, trapping them in cycles of relational dependency on sensitive teachers and academic disadvantage.

Conversely, PE's transformative potential lies in two key areas. (1) *Intrinsic Motivation*: Many students with SEN-ESD describe PE as their favorite subject. Some students' drive for movement provides a pathway for engagement. (2) *Social-Emotional Skill-Building*: Curricular mandates (3, 4) position PE as a platform for growth—*if* teachers employ sensitivity. Without accommodations and explicit training in autonomous conflict resolution, students can remain trapped in cycles of relational and academic disadvantage.

### 4.2 Strengths and limitations

This study has several notable *strengths*. *First*, the category TSR emerged *inductively* from both perspectives, highlighting its importance to the participants themselves. *Second*, our *pioneering dual-perspective design* bridges a critical gap in TSR research: to our knowledge, this is the first study to openly interview both students with SEN-ESD and their teachers, *providing student voices often neglected in prior work*. *Third*, *Grounded Theory* ensured methodological robustness, uncovering implicit beliefs and latent constructs that standardized quantitative tools like the Teacher-Student Relationship Interview (TSI) miss. *Fourth*, the study offers *innovative conceptual contributions* by centering participant-driven definitions, revealing previously untheorized attributes of affective TSR quality. These findings were deductively linked to Pianta's dimensions of closeness and conflict dimensions, while the dependency dimension emerged as irrelevant for students classified with externalizing behaviors—a novel qualitative insight. These strengths advance nuanced, context-sensitive insights into dyadic TSRs in inclusive PE.

This study has *four limitations*. *First*, generalizing results into patterns risks oversimplifying nuanced interview data (28). To address this, we integrated detailed case studies and analyzed each conflict-laden situation through memoing to preserve narrative richness, while an interdisciplinary Grounded Theory group validated interpretations—ensuring findings remain grounded in participants' experiences while offering accessible syntheses. *Second,* while focusing on formally diagnosed students risks overgeneralization, the central finding—the critical importance of close TSR and teacher sensitivity—may be applicable to many children with similar support needs, including those who are undiagnosed or fall under broader conceptualizations of emotional and social health. *Third,* constructs like “functional conflict resolution” were conceptualized along shared dimensions despite varying participant definitions. To align with Grounded Theory principles, we prioritized empirically reconstructed subjective perspectives, foregrounding participants' own meanings over imposed normative frameworks.

*Fourth*, while this study broadly sampled SEN-ESD students and PE teachers rather than analyzing predefined dyads, the identification of consistent patterns across participants until theoretical saturation suggests findings may be transferable to dyadic contexts.

### 4.3 Future research directions

While this study advances understanding of TSRs in inclusive PE, critical gaps remain. Qualitative research foregrounding the experiences of students with SEN-ESD in PE remains strikingly scarce. Prior to this study, only one study (31) had centered student voices in mainstream contexts—leaving a significant gap regarding SEN-ESD experiences. Furthermore, cross-disciplinary TSR research has historically relied on teacher-centric, correlational designs predominantly focusing on younger children (1), leaving adolescents' relational dynamics and qualitative designs underexplored.

Four priorities emerge: (*1*) *Perceptual discrepancies*: Future research should test whether the divergent interpretive logics identified in this study mediate teacher-student rating discrepancies observed in quantitative studies (21, 22). (*2*) *Beyond TSR*: Given that perceptual discrepancies likely extend beyond TSRs, dual-perspective qualitative research should examine potential mismatches in core topics like inclusion, fairness, recognition, and autonomy. Such work is essential for developing equitable practices that reflect student-identified needs rather than institutional assumptions. (*3*) *Measurement alignment*: Future quantitative studies should validate scales integrating context-specific practices—individualized agreements, proactive conflict resolution, and structured autonomy—to refine Pianta's (20) closeness dimension in the STRS from a student perspective. (*4*) *Contextual and longitudinal insights*: Comparative studies across mainstream and special education settings should examine how institutional cultures shape relational practices. Longitudinal designs could track dyadic trust evolution, while case studies of student-teacher pairs might reveal micro-level negotiation processes.

## 5 Conclusion

This study represents provides the first qualitative investigation of teacher-student relationships (TSR) in inclusive Physical Education (PE) for students with special educational needs in their emotional and social development (SEN-ESD), capturing dual perspectives from students and teachers. Three key contributions advance understanding of relational dynamics in inclusive contexts: *First*, the perceived quality of *TSR is intrinsically tied to teacher sensitivity*. Close TSRs (17) manifested when educators established individualized agreements while differentiating behavioral expectations for SEN-ESD students. These practices created relational “safer spaces”, enabling students to employ *subjectively functional emotion regulation strategies* (26, 27) amid perceived frequent PE conflicts—particularly important given SEN-ESD students' possible struggles with self-regulation (5).

*Second*, *subjective-normative discrepancies* characterize emotion regulation strategies: some students experiencing a conflictual TSR (20) described normatively dysfunctional strategies (e.g., aggression) as subjectively functional, often rationalizing them through moral reasoning frameworks of reciprocity. Recognizing these divergent implicit interpretive logics as students' subjective reality is important; reciprocal dialogue can foster trust and disrupt cycles of marginalization.

*Third*, teachers' *ad hoc attachment* roles can *disrupt negative relational cycles* and counteract insecure attachment patterns common among SEN-ESD students (15), *if* sensitivity guides differentiated expectations. Sustaining this requires explicit *teacher training* in attachment-sensitive pedagogy to understand deviant behavior and teachers' own relationship representations (17), which otherwise act as self-fulfilling prophecies. Concurrently, *training students* in *autonomous conflict-resolution* can empower social-emotional growth, disrupting marginalization cycles. PE's unique potential lies in harnessing students' intrinsic motivation for PE alongside curricular mandates for social-emotional skill development (3, 4).

*Methodologically*, these findings necessitate *revising relational assessment tools* like the Teacher-Student-Relationship Interview (TSI) to integrate e.g., individualized agreements as a core component of the closeness dimension. *Limitations* including non-dyadic sampling highlight the need for longitudinal and comparative research in mainstream and special education to. Dual-perspective designs exploring participatory interactions could further address gaps in understanding equitable practices.

Ultimately, this work positions *PE as a relational microcosm* where inclusion is negotiated daily. By bridging student and teacher voices, it challenges deficit narratives and offers a roadmap for leveraging PE's unique potential—a *space where movement, emotion, and trust can intersect to foster growth*.

## Data Availability

The datasets presented in this article are not readily available because the raw interview data contain sensitive information that could compromise participant privacy and confidentiality. The interview protocol and analytical codebook are available from the corresponding author upon reasonable request. Requests to access the datasets should be directed to leefke.brunssen@uni-bielefeld.de.

## Footnotes

^1^We retain SEN-ESD (‘Förderschwerpunkt emotionale und soziale Entwicklung’) given its alignment with German legal frameworks (Conference of the Ministers of Education and Cultural Affairs guidelines) and contextual specificity to the educational system studied. We acknowledge ongoing debates about translating this SEN area—where anglophone labels like Emotional/Behavioral Disorder (EBD) or Social/Emotional/Mental Health (SEMH) are sometimes adopted.

^2^EBP is a literature-established term. We use "problem" critically, recognizing it reflects normative judgments that risk pathologizing students.

^3^Critically, the *labelling-resource dilemma* lies at the heart of inclusive education: diagnostic labels (e.g., SEN-ESD) are necessary to allocate specialized support, yet overreliance on them risks pathologizing students through deficit-oriented narratives.

^4^Emphasized words or word parts are written in capital letters.

^5^A popular ball game in Germany similar to rounders or kickball.
